# Use of whole genome sequencing to identify low‐frequency mutations in SARS‐CoV‐2 patients treated with remdesivir

**DOI:** 10.1111/irv.13179

**Published:** 2023-09-26

**Authors:** Kuganya Nirmalarajah, Winfield Yim, Patryk Aftanas, Angel X. Li, Altynay Shigayeva, Lily Yip, Zoe Zhong, Allison J. McGeer, Finlay Maguire, Samira Mubareka, Robert Kozak

**Affiliations:** ^1^ Sunnybrook Research Institute Toronto Ontario Canada; ^2^ Department of Laboratory Medicine and Pathobiology University of Toronto Toronto Ontario Canada; ^3^ Shared Hospital Laboratory Toronto Ontario Canada; ^4^ Sinai Health System Toronto Ontario Canada; ^5^ Faculty of Computer Science Dalhousie University Halifax Nova Scotia Canada; ^6^ Department of Community Health & Epidemiology Dalhousie University Halifax Nova Scotia Canada

**Keywords:** antiviral resistance, remdesivir, SARS‐CoV‐2, whole genome sequencing

## Abstract

**Background:**

Remdesivir (RDV) has been shown to reduce hospitalization and mortality in COVID‐19 patients. Resistance mutations caused by RDV are rare and have been predominantly reported in patients who are on prolonged therapy and immunocompromised. We investigate the effects of RDV treatment on intra‐host SARS‐CoV‐2 diversity and low‐frequency mutations in moderately ill hospitalized COVID‐19 patients and compare them to patients without RDV treatment.

**Methods:**

From March 2020 to April 2022, sequential collections of nasopharyngeal and mid‐turbinate swabs were obtained from 14 patients with and 30 patients without RDV treatment. Demographic and clinical data on all patients were reviewed. A total of 109 samples were sequenced and mutation analyses were performed.

**Results:**

Previously reported drug resistant mutations in nsp12 were not identified during short courses of RDV therapy. In genes encoding and surrounding the replication complex (nsp6‐nsp14), low‐frequency minority variants were detected in 7/14 (50%) and 18/30 (60%) patients with and without RDV treatment, respectively. We did not detect significant differences in within‐host diversity and positive selection between the RDV‐treated and untreated groups.

**Conclusions:**

Minimal intra‐host variability and stochastic low‐frequency variants detected in moderately ill patients suggests little selective pressure in patients receiving short courses of RDV. The barrier to RDV resistance is high in patients with moderate disease. Patients undergoing short regimens of RDV therapy should continue to be monitored.

## 1 INTRODUCTION

The SARS‐CoV‐2 pandemic has resulted in millions of deaths, and while vaccines have been shown to have high effectiveness in preventing severe disease,^1^ there have been breakthrough infections, and some populations remain at risk for severe disease. SARS‐CoV‐2 specific small molecule inhibitors offer a promising means of reducing hospitalizations and mortality. Remdesivir (RDV) is a nucleotide analog that is administered as a prodrug, and subsequently converted by the host cell into the active triphosphate form to inhibit viral replication^2^ and has maintained activity against all variants that have emerged up to Omicron.^3^ Therapeutic efficacy demonstrated by increased survival has been noted in hospitalized and out‐patient trials.^4^, ^5^ Resistance conferring mutations to RDV are very rare based on a survey of SARS‐CoV‐2 genomes available in GISAID^6^ and has been primarily reported in immunocompromised patients who are on prolonged therapy.^7^, ^8^ Additionally, a recent report highlighted an immunocompromised patient who had rebound infections and multiple courses of RDV, but no resistance mutations were identified.^9^ Overall, there is a paucity of data on patients with serial sample collections over the course of their treatment making it difficult to determine when, or if, resistance mutations arise, and at what frequency.

Inadequate therapy can be a driver for the selection of drug resistance. While mutations conferring resistance to RDV have been identified in patients who received prolonged therapies for SARS‐CoV‐2,^7^, ^10^ it remains unclear if hospitalized individuals who received shorter courses of therapy are likely to develop mutations that confer resistance to RDV. High‐throughput sequencing is a method for detecting low‐frequency variants that emerge following the administration of antiviral therapy.^11^, ^12^ In this study, we collected serial samples from patients treated with RDV, including those who had shortened courses of therapy. Novel polymorphisms identified as low‐frequency variants were analyzed for their potential resistance profiles and tested for selection.

## 2 MATERIALS & METHODS

### 2.1 Patients and sample collection

Serial samples obtained from hospitalized adult patients with laboratory‐confirmed COVID‐19 were recruited from five hospital sites in the greater Toronto area. Patients were prospectively recruited by the Toronto Invasive Bacterial Disease Network (TIBDN) from March 2020 to April 2022. Informed consent was obtained and all participating hospitals granted research ethics approval by their respective ethics boards (REB #2218). Demographic and clinical data were obtained from electronic medical record review and patient interview. Serial nasopharyngeal or mid‐turbinate samples were collected starting from date of study enrollment until recovery, refusal, or death.

### 2.2 Whole genome sequencing (WGS)

The ARTIC SARS‐CoV‐2 protocol (https://artic.network/ncov-2019) was used for amplicon generation. Sequencing was performed using the methods outlined.^13^, ^14^ Briefly, nucleic acid extraction was performed^15^ and cDNA was generated by the ARTIC protocol. After cDNA synthesis, two multiplex PCR tiling reactions were prepared using the ARTIC V3, V4, and V4.1 primers (depending on sampling date). cDNA was combined with Q5 High‐Fidelity 2X Master Mix (NEB, USA). To mix #1, 10 μM of ARTIC primer pool #1 were added and 10 μM of ARTIC primer pool #2 were added to mix #2. Cycling was then performed as follows: 98°C for 30 s followed by 35 cycles of 98°C for 15 s and 65°C for 5 min. Following this reaction, products were combined, and a clean‐up with AMPure XP beads was performed (Beckman Coulter, USA). The quantity of amplicons was measured with the Qubit 4.0 fluorometer using the 1X dsDNA HS Assay Kit (Thermo Fisher Scientific, USA). The sequencing libraries were prepared using the Nextera DNA Flex Prep kit (Illumina, USA) as per manufacturer's instructions. Paired‐end (2 × 150 bp) sequencing was performed on a MiniSeq with a 300‐cycle reagent kit (Illumina, USA).

### 2.3 Bioinformatic analysis

Short‐read sequence processing, genome assembly, and Pangolin lineage assignment were done using the SIGNAL v1.5.6 (SARS‐CoV‐2 Illumina GeNome Assembly Line) pipeline (https://github.com/jaleezyy/covid-19-signal).^14^ Within SIGNAL, the consensus genome sequences are generated based on the SARS‐CoV‐2 Wuhan/Hu‐1/2019 reference genome (MN908947.3) using BWA‐MEM^16^ v0.7.17. PANGO^17^ lineages were assigned using pangolin^18^ v3.1.17 with PangoLEARN 2022‐01‐20 and coverage statistics were calculated with BEDTools^19^ v2.26.0. Sequences with less than 75% genome completeness and 500 times coverage depth were excluded from subsequent analyses. Annotated amino acid mutations were predicted by Breseq v035.6 using cut‐offs of 0.05 for minimum polymorphism frequency and 2 for polymorphism minimum coverage.^20^ Results were visualized using the pandas^21^ v1.5.0, matplotlib^22^ v3.5.3, and seaborn^23^ v0.11.2 packages via Python v3.10.7.

### 2.4 Phylogenetic analysis

Transition (Ti) and transversion (Tv) substitutions, deletions, and insertions were tabulated from Breseq results. Hypothesis Testing using Phylogenies (HyPhy) v2.5.42 was used for testing signals of positive selection^24^ in the ORF1ab gene on RDV‐treated sequences. Prior to selection analysis, Virulign^25^ v1.0.1 was used to generate codon alignments of all sequences with the SARS‐CoV‐2 Wuhan/Hu‐1/2019 (MN908947.3) ORF1ab as the reference. A HyPhy inferred tree was generated and phylogenies were labeled using the Phylotree Widget. Excess stop codons were removed using HyPhy before performing selection analysis. The adaptive branch‐site random effects likelihood (aBSREL)^26^ method from HyPhy was used to test for evidence of positive selection among the foreground RDV branches relative to the untreated sequences.

### 2.5 Statistical analysis

Patient demographics and clinical characteristics were compared between the RDV‐treated and untreated groups using Welch's *t*‐test for continuous variables and Pearson's Chi‐Square or Fisher's exact tests for categorical variables. Statistical testing and plotting were performed using RStudio v1.4.1717 with the dplyr^27^ v1.0.10 and ggplot2^28^ v3.3.6 packages. The Mann Whitney U test was used to compare the distribution of mutation counts, and transition and transversion mutation types between patients with and without RDV treatment. The Chi‐Square test was used to assess the difference between the Ti/Tv ratios in the RDV‐treated and untreated groups. Statistical significance was determined based on a *p*‐value of less than 0.05.

## 3 RESULTS

### 3.1 Cohort description

**FIGURE 1:**
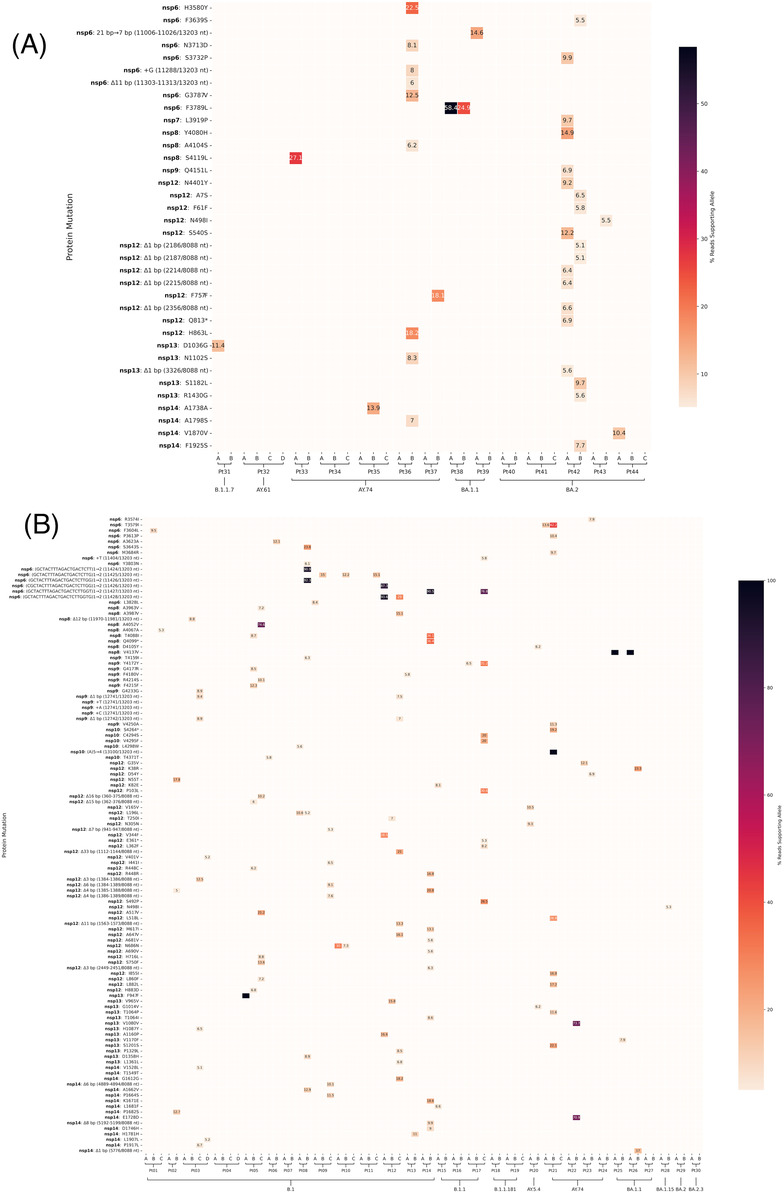
Mutation profile across nsp6‐nsp14 for 35 serial swabs collected from RDV‐treated patients (A) and 74 collected from untreated patients (B). Alphabetical ordering of patient serial samples represents collection date order. Mutations were analyzed and called against the reference sequence with the Breseq pipeline. Low‐frequency minority mutations were retained and percentages are shown. Majority mutations conserved among lineages and within individuals are excluded. Default parameters of Breseq including a minimum 5% polymorphism frequency filter and a polymorphism minimum coverage of 2 were used.

In total, 44 patients who were hospitalized from March 2020 to April 2022 and had at least two SARS‐CoV‐2 positive nasal samples were included in the study. Patient demographic and clinical characteristics are shown in Table 1. Fourteen (31.8%) patients received RDV for a median duration of 4 days (IQR, 2.5–9). Patients were sampled a median of 2 (IQR, 2–3) times following commencement of therapy and collection times are shown in Table 2. Samples were subjected to WGS if SARS‐CoV‐2 RT‐PCR cycle threshold values for the E‐gene target were less than 30 (Tables S1 and S2). A total of 109 complete genomes were generated from 44 patients. Patients were infected with early B.1, B.1.1, and B.1.1.181 SARS‐CoV‐2 lineages and variants of concern (VOCs) including Alpha, Delta, and Omicron (Figure 1). No patients received additional antivirals in hospital following RDV treatment.

**TABLE 1:** Patient demographics and clinical characteristics of cohort.

	Total (*n* = 44)	Remdesivir (*n* = 14)	No remdesivir (*n* = 30)	*p*‐Value
Median age, years (IQR)	75.5 (62.5–85)	78.5 (59.2–82)	74 (63–87.2)	0.72
≥65 years	29 (65.9%)	9 (64.3%)	20 (66.7%)	1
≤64 years	15 (34.1%)	5 (35.7%)	10 (33.3%)	1
Male sex	27 (61.4%)	7 (50%)	20 (66.7%)	0.47
Median duration of hospitalization (IQR)	14 (10.8–25.2)	11 (10.25–15)	16 (11.2–34.2)	0.38
Median duration of RDV treatment (IQR)		4 (2.5–9)		
ICU admission	2 (4.5%)	2 (14.3%)	0	0.10
Mortality	7 (15.9%)	0	7 (23.3%)	0.08
Obesity	2 (4.5%)	1 (7.1%)	1 (3.3%)	0.54
Diabetes	14 (31.8%)	6 (42.9%)	8 (26.67%)	0.32
Cardiac disease	17 (38.6%)	6 (42.9%)	11 (36.7%)	0.75
Vascular disease	26 (59.1%)	9 (64.3%)	17 (56.7%)	0.88
Pulmonary disease	16 (36.4%)	6 (42.9%)	10 (33.3%)	0.78
Renal disease	8 (18.2%)	4 (28.6%)	4 (13.3%)	0.24
Cancer	8 (18.2%)	3 (21.4%)	4 (13.3%)	0.70
Immunosuppressed	5 (11.4%)	3 (21.4%)	2 (6.7%)	0.35

Abbreviations: ICU, intensive care unit; IQR, interquartile range; RDV, remdesivir.

**TABLE 2:** Sampling time points collected for patients treated with RDV.

Patient	Lineage	Length of hospital stay (days)	Time between symptom onset and RDV initiation (days)	Duration of RDV treatment (days)	Days of sampling relative to RDV start (Day 0)																
					−5	−4	−3	−2	−1	0	1	2	3	4	5	6	7	8	9	10	11
Pt31	B.1.1.7 (Alpha)	15	5	8							7										
Pt32	AY.61 (Delta)	12	1	9							11.5										
Pt33	AY.74 (Delta)	9	9	9																	
Pt34	AY.74 (Delta)	11	4	9							2										
Pt35	AY.74 (Delta)	11	8	10									.4						2		
Pt36	AY.74 (Delta)	83	3	4									5								
Pt37	AY.74 (Delta)	8	11	10								N/A									
Pt38	BA.1.1 (Omicron)	11	4	4							15.1										
Pt39	BA.1.1 (Omicron)	15	2	2																	
Pt40	BA.2 (Omicron)	16	2	4																	
Pt41	BA.2 (Omicron)	10	4	1							3		23.5								
Pt42	BA.2 (Omicron)	55	2	2																	
Pt43	BA.2 (Omicron)	11	4	4							6										
Pt44	BA.2 (Omicron)	4	5	2							3		8								

Abbreviation: RDV, remdesivir.

### 3.2 Drug resistance mutations were not identified by WGS in RDV‐treated patient cohort

Multiple mutations have been reported that confer varying levels of resistance to RDV in the viral replication complex.^6^ We examined 35 serial collections obtained from 14 RDV‐treated patients. The consensus genomes were examined and neither E802D, a mutation in nsp12 associated with treatment failure,^7^ nor other known resistance mutations were not detected as a consensus mutation in any patient samples regardless of the duration of RDV therapy. In parallel, we analyzed 74 serial samples collected from 30 patients who did not receive RDV therapy and did not identify any drug‐resistance mutations.

### 3.3 Low‐frequency variants identified during treatment

The highly sensitive sequencing platform allows for detection and quantification of low‐frequency variants that would not be identified in the consensus sequence. Work by other groups suggests that only mutations present at >15% were likely to represent viral adaptation in the face of selective drug pressure, thus we used a similar cut off for classifying low‐frequency mutations under this threshold.^29^ Low‐frequency minority variants within the replication complex and surrounding genes (nsp6‐nsp14) were identified in 7/14 (50%) of RDV‐treated patients (Figure 1A). These low‐frequency variants were unique instances and not repeatedly detected across patients. Minority variants were identified in 18/30 (60%) of patients without RDV treatment (Figure 1B). Low‐frequency variants identified in early time points did not persist in subsequent collections and did not become fixed in either group. Additionally, no low‐frequency variants were observed at E802 or V166, both positions associated with RDV resistance in patients.^7^, ^10^ Low‐frequency variants were also observed in other regions of the genome in both treatment groups (Figure S1a and b).

### 3.4 Within‐host variability detected in RDV‐treated and untreated patient cohorts

**FIGURE 2:**
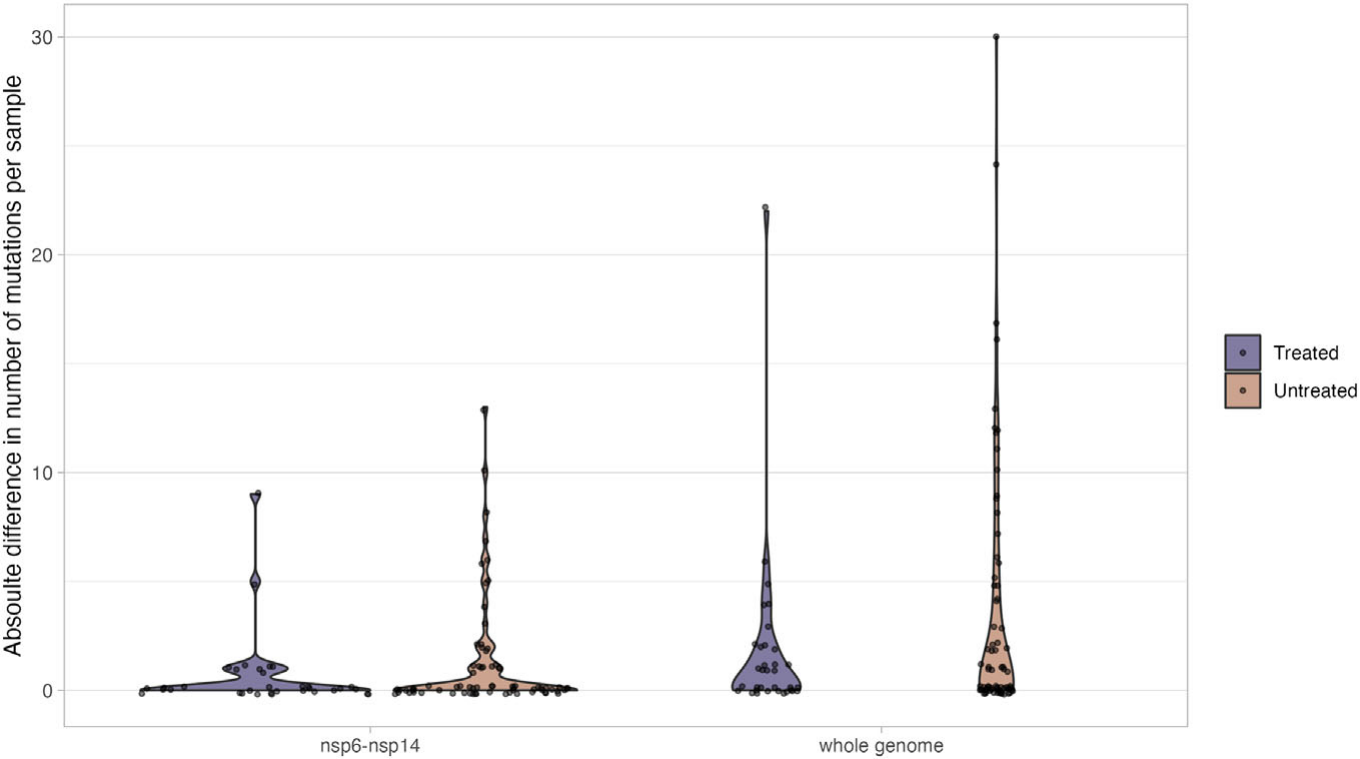
Absolute difference in number of total consensus and low‐frequency mutations (including SNPs, amino acid insertions, amino acid deletions, and complex mutations) relative to the first examined sample of each patient within nsp6‐nsp14 and the whole genome. The Mann Whitney U test was used to calculate *p‐*values comparing distributions between RDV‐treated and untreated groups.

We investigated intra‐host viral diversity for patients treated with and without RDV therapy. Changes in the total number of amino acid mutations identified by Breseq, including synonymous and nonsynonymous single nucleotide polymorphisms (SNPs), insertions, deletions, and complex substitutions across samples from different time points can reveal intra‐host viral diversity. Intra‐host variability was detected in nsp6‐nsp14 across serial collections in 10/14 (71.4%) patients treated with RDV (Figure 1A). However, there were no changes in the consensus sequences of serial collections in 4/14 (28.6%) patients, indicating no intra‐host diversity. Within‐host diversity in nsp6‐nsp14 was detected in 20/30 (66.7%) patients without RDV therapy (Figure 1B). There were 13/14 (92.8%) and 27/30 (90.0%) patients in the RDV‐treated (Figure S1a) and untreated (Figure S1b) groups, respectively, where intra‐host variation across serial collections were detected throughout the whole genome. Total within‐host diversity was also compared among the RDV‐treated and untreated groups by investigating the absolute difference in the number of total mutations per sample relative to the first analyzed serial collection (Figure 2). Within‐host diversity between the RDV‐treated and untreated groups in the nsp6‐nsp14 region or the whole genome were not significantly different based on the Mann Whitney U test.

### 3.5 Within‐host variability and selection processes are minimal in RDV‐treated and untreated patients

**FIGURE 3:**
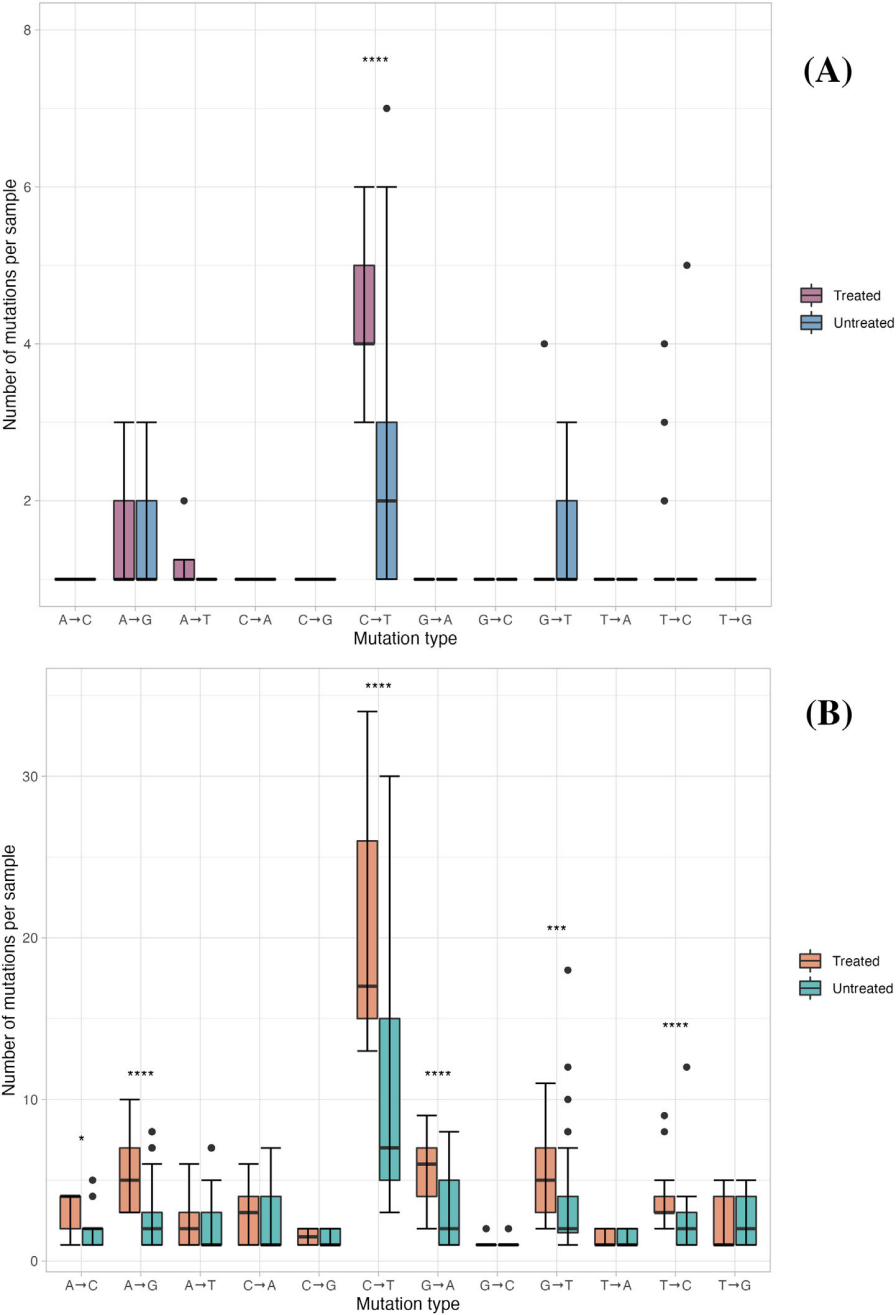
Boxplot summary of types of mutations representing transitions and transversions events in the RDV‐treated and untreated groups within nsp6‐nsp14 (A) and the whole genome (B). Points represent individual collections with counts of each substitution type for 35 samples from RDV‐treated patients and 74 samples from patients without RDV treatment. The Mann Whitney U test was used to calculate *p‐*values comparing each mutation distribution between RDV‐treated and untreated groups.

Transition (Ti) and transversion (Tv) events were examined across the whole genome for patients treated with and without RDV therapy. Transitions are substitutions interchanging bases of the same ring structure while transversions are interchanges of purine bases for pyrimidine bases. The total number of all transition and transversion events from each sample from all patients among the RDV‐treated and untreated group were compared for the nsp6‐nsp14 region (Figure 3A) and the whole genome (Figure 3B). C → T Ti were the most common type of substitutions in both groups. Prior to correcting for phylogenies, significant differences in the number C → T mutations in the nsp6‐nsp14 region (Figure 3A) and A → G, C → T, G → A, G → T, and T → C (Figure 3B) mutations in the whole genome between the RDV‐treated and untreated group were observed. However, these differences can be attributed to the varying makeup of lineages in each treatment group, as the untreated group contains mostly early lineages. In the RDV‐treated group, 1165 Ti and 805 Tv were identified within the whole genome, giving a Ti/Tv ratio of 1.45. There were 1245 Ti and 664 Tv in the untreated group within the whole genome with a Ti/Tv ratio of 1.88. When comparing the Ti/Tv ratios among both groups, no significant difference was observed. Furthermore, we identified 144 small and mid‐size deletions across the whole genome in the 14 RDV‐treated patients of which 18 were low frequency (Figure S1a). 142 deletions were detected in the 30 patients without RDV treatment with 43 low‐frequency deletions (Figure S1b). Deletions are denoted on heatmap figures by “Δ bp coding.” Most deletions detected were found in the consensus sequences of samples and are characteristic of VOCs particularly the Delta and Omicron lineages.

To determine whether selective pressures were exerted in the RDV‐treated group, we tested for episodic positive selection while correcting for the phylogenetic structure of our samples. Codon alignments for ORF1ab were generated for all sequences and the aBSREL model^26^ from HyPhy^24^ was applied to an RDV‐treatment labeled phylogeny generated by the Phylotree Widget. No evidence of episodic diversifying positive selection was identified in the ORF1ab gene in the foreground RDV branches relative to the untreated branches. Together, this suggests that evolutionary processes such as positive selection, replicase complex adaptation, and resistance emergence are minimal with RDV treatment.

## 4 DISCUSSION

In this study, WGS was used to identify low‐frequency mutations in patients with serial collections during RDV therapy. Low‐frequency variants were not detected in E802, D484, V166, or at any of the other positions reported previously.^6^, ^7^, ^8^, ^9^ Overall, RDV did not result in the generation of low‐frequency drug‐resistance mutations in the short term. In RDV‐treated patients, within nsp6‐nsp14, we did not detect the persistence of low‐frequency variants across time points from the same individuals or recurring variants at the same loci among different RDV patients. Additionally, within nsp6‐nsp14, accumulation of de novo low, intermediate, or fixed variants throughout the course of infection in either the RDV‐treated or untreated groups was not observed. Together, this suggests that low and intermediate frequency variants detected in samples from RDV patients arose sporadically and are likely random mutations rather than drug‐resistance mutations. In contrast to our findings, Gandhi et al. reported the emergence and temporal increase in allele frequency of the de novo nsp12 E802D mutation in an RDV‐treated immunocompromised patient after Day 7 of therapy.^7^ To date, there have only been case reports of antiviral resistance being identified, and no one has performed serial sampling in a larger set of patients on RDV therapy with a comparison group.

Similar to Heyer et al.,^10^ we saw very little increase in variant allele frequencies and minimal intra‐host diversity in the samples from RDV‐treated patients. However, they reported increased genomic variability and nine novel nucleotide variants (including two in the RdRp) that reached apparent fixation in a single patient with follicular lymphoma suffering from prolonged infection and was treated with two courses of RDV.^10^ Such observations suggest that RDV treatment can lead to increased intra‐host genomic diversity and emergence of fixed mutations in immunodeficient hosts with prolonged infections. Furthermore, Heyer et al. argue that the emergence of novel variants cannot be attributed to prolonged infection alone as similar patients given other treatments such as anti‐inflammatories resulted in sporadic, transient, and lower frequency variants. Existing studies suggest that within‐host dynamics arise stochastically and that mutations that are advantageous to the virus may exist at low‐frequency and emerge under selective pressure.^10^ Our data suggest that if this is the case, strong selection is not present in the early phase of treatment. We speculate that minimal intra‐host diversity and the emergence of low‐frequency variants arise stochastically due to genetic drift and not from RDV selective pressures. Other studies in persistently infected immunocompromised patients have also failed to identify resistance mutations to RDV,^30^ although interestingly, mutations that reduce monoclonal antibody neutralization efficacy arise more easily.

Our study is novel as it focused on patients who received less than 10 days of therapy and indicates that this does not select for resistant viruses in hospitalized patients. While it cannot be discounted that mutations arose at later time points, this seems unlikely as all RDV‐treated patients recovered and none had prolonged infection. This is indirectly supported by the fact that RDV has been used as 3‐day treatment for non‐hospitalized patients and has not resulted in documented resistance emerging.^5^ Collectively, these findings suggest that the barrier to resistance is high and that host‐immunosuppression is needed for the development of resistance. We highlight these effects in moderately ill patients with shorter‐term infection without severe immunocompromise. This underscores the relative safety of RDV against the emergence of drug resistant mutations in such patients. Our study had several limitations, including the small cohort size, and that only patient samples with sufficiently high viral load (e.g., Ct < 30) were suitable for sequencing. The phylogenetic makeup of our treatment groups also varied. We did attempt to control for the phylogenetic structure while testing for positive selection; however, this will still reduce our statistical power.

Here, we highlight the effects of short courses of RDV on low‐frequency mutations and SARS‐CoV‐2 intra‐host variability in patients with moderate disease. Resistance mutations were not detected within the replication complex and intra‐host diversity remains minimal in RDV‐treated patients. This suggests that the barrier to resistance is high, likely requiring prolonged infection and immunosuppression of the host. Future improvements to whole‐genome sequencing assays are needed to interrogate samples with low viral loads and improve surveillance for antiviral resistance mutations.

## AUTHOR CONTRIBUTIONS

**Kuganya Nirmalarajah**: Formal analysis; investigation; methodology; visualization; writing—original draft preparation; writing—review and editing. **Winfield Yim**: Formal analysis; visualization. **Patryk Aftanas**: Methodology. **Angel X. Li**: Project administration. **Altynay Shigayeva**: Data curation. **Lily Yip**: Project administration. **Xi Zoe Zhong**: Project administration. **Allison J. McGeer**: Resources; writing—review and editing. **Finlay Maguire**: Supervision; writing—review and editing. **Samira Mubareka**: Funding acquisition; resources; supervision; writing—review and editing. **Robert Kozak**: Conceptualization; supervision; writing—original draft preparation; writing—review and editing.

## CONFLICT OF INTEREST STATEMENT

The authors report no conflicts of interest for this work.

### PEER REVIEW

The peer review history for this article is available at https://www.webofscience.com/api/gateway/wos/peer-review/10.1111/irv.13179.

## ETHICS STATEMENT

Informed consent was obtained from patients in this study. All participating hospitals granted research ethics approval by their respective ethics boards (REB #2218).

## Supporting information


**Figure S1a** Mutation profile across the whole genome for 35 serial swabs collected from remdesivir treated patients (A) and 74 collected from untreated patients (B). Serial samples are labeled sequentially by date. Mutations were analyzed and called against the reference sequence with the Breseq pipeline. Low frequency minority mutations were retained and percentages are shown. Majority mutations conserved among lineages and within individuals are excluded. Default parameters of Breseq including a minimum 5% polymorphism frequency filter and a polymorphism minimum coverage of 2 were used.

**Figure S1b** Mutation profile across the whole genome for 35 serial swabs collected from remdesivir treated patients (A) and 74 collected from untreated patients (B). Serial samples are labeled sequentially by date. Mutations were analyzed and called against the reference sequence with the Breseq pipeline. Low frequency minority mutations were retained and percentages are shown. Majority mutations conserved among lineages and within individuals are excluded. Default parameters of Breseq including a minimum 5% polymorphism frequency filter and a polymorphism minimum coverage of 2 were used.

**Table S1** Cycle threshold for the SAR‐CoV‐2 envelope gene from nasal samples collected from RDV‐treated patients.
**Table S2** Cycle threshold for the SAR‐CoV‐2 envelope gene from nasal samples collected from patients without RDV treatment.

## Data Availability

The data that support the findings of this study are available from the corresponding author upon request.

## References

[1] Lopez Bernal J , Andrews N , Gower C , et al. Effectiveness of covid‐19 vaccines against the B.1.617.2 (delta) variant. N Engl J Med. 2021;385(7):585‐594. doi:10.1056/NEJMoa2108891 34289274 PMC8314739

[2] Yin W , Mao C , Luan X , et al. Structural basis for inhibition of the RNA‐dependent RNA polymerase from SARS‐COV‐2 by remdesivir. Science. 2020;368(6498):1499‐1504. doi:10.1126/science.abc1560 32358203 PMC7199908

[3] Vangeel L , Chiu W , De Jonghe S , et al. Remdesivir, Molnupiravir and Nirmatrelvir remain active against SARS‐COV‐2 omicron and other variants of concern. Antiviral Res. 2022;198:105252. doi:10.1016/j.antiviral.2022.105252 35085683 PMC8785409

[4] Ali K , Azher T , Baqi M , et al. Remdesivir for the treatment of patients in hospital with covid‐19 in Canada: a randomized controlled trial. Can Med Assoc J. 2022;194(7):E242‐E251. doi:10.1503/cmaj.211698 35045989 PMC8863204

[5] Gottlieb RL , Vaca CE , Paredes R , et al. Early remdesivir to prevent progression to severe covid‐19 in outpatients. N Engl J Med. 2022;386(4):305‐315. doi:10.1056/NEJMoa2116846 34937145 PMC8757570

[6] Focosi D , Maggi F , McConnell S , Casadevall A . Very low levels of remdesivir resistance in SARS‐COV‐2 genomes after 18 months of massive usage during the covid19 pandemic: a Gisaid exploratory analysis. Antiviral Res. 2022;198:105247. doi:10.1016/j.antiviral.2022.105247 35033572 PMC8755559

[7] Gandhi S , Klein J , Robertson AJ , et al. De novo emergence of a remdesivir resistance mutation during treatment of persistent SARS‐COV‐2 infection in an immunocompromised patient: a case report. Nat Commun. 2022;13(1):1547. doi:10.1038/s41467-022-29104-y 35301314 PMC8930970

[8] Martinot M , Jary A , Fafi‐Kremer S , et al. Emerging RNA‐dependent RNA polymerase mutation in a remdesivir‐treated B‐cell immunodeficient patient with protracted coronavirus disease 2019. Clin Infect Dis. 2020;73(7):e1762‐e1765. doi:10.1093/cid/ciaa1474 PMC7543308 32986807

[9] Martinez MA , Chen T‐Y , Choi H , et al. Extended REMDESIVIR infusion for persistent coronavirus disease 2019 infection. Open Forum Infect Dis. 2022;9(8):ofac382. doi:10.1093/ofid/ofac382 36039098 PMC9384609

[10] Heyer A , Günther T , Robitaille A , et al. Remdesivir‐induced emergence of SARS‐COV2 variants in patients with prolonged infection. Cell Rep Med. 2022;3(9):100735. doi:10.1016/j.xcrm.2022.100735 36075217 PMC9378267

[11] Ji H , Kozak RA , Biondi MJ , et al. Next generation sequencing of the hepatitis C virus NS5B gene reveals potential novel S282 drug resistance mutations. Virology. 2015;477:1‐9. doi:10.1016/j.virol.2014.12.037 25600207

[12] Tzou PL , Ariyaratne P , Varghese V , et al. Comparison of an in vitro diagnostic next‐generation sequencing assay with sanger sequencing for HIV‐1 genotypic resistance testing. J Clin Microbiol. 2018;56(6):e00105‐e00118. doi:10.1128/JCM.00105-18 29618499 PMC5971553

[13] Kotwa JD , Jamal AJ , Mbareche H , et al. Surface and air contamination with severe acute respiratory syndrome coronavirus 2 from hospitalized coronavirus disease 2019 patients in Toronto, Canada, march–may 2020. J Infect Dis. 2021;225(5):768‐776. doi:10.1093/infdis/jiab578 PMC8767887 34850051

[14] Nasir JA , Kozak RA , Aftanas P , et al. A comparison of whole genome sequencing of SARS‐COV‐2 using amplicon‐based sequencing, random hexamers, and bait capture. Viruses. 2020;12(8):895. doi:10.3390/v12080895 32824272 PMC7472420

[15] Feld JJ , Kandel C , Biondi MJ , et al. Peginterferon lambda for the treatment of outpatients with covid‐19: a phase 2, placebo‐controlled randomised trial. Lancet Respir Med. 2021;9(5):498‐510. doi:10.1016/S2213-2600(20)30566-X 33556319 PMC7906707

[16] Li H , Durbin R . Fast and accurate short read alignment with burrows‐wheeler transform. Bioinformatics. 2009;25(14):1754‐1760. doi:10.1093/bioinformatics/btp324 19451168 PMC2705234

[17] Rambaut A , Holmes EC , O'Toole Á , et al. A dynamic nomenclature proposal for SARS‐COV‐2 lineages to assist genomic epidemiology. Nat Microbiol. 2020;5(11):1403‐1407. doi:10.1038/s41564-020-0770-5 32669681 PMC7610519

[18] O'Toole Á , Scher E , Underwood A , et al. Assignment of epidemiological lineages in an emerging pandemic using the pangolin tool. Virus Evolution. 2021;7(2):veab064. doi:10.1093/ve/veab064 34527285 PMC8344591

[19] Quinlan AR , Hall IM . BEDTools: a flexible suite of utilities for comparing genomic features. Bioinformatics. 2010;26(6):841‐842. doi:10.1093/bioinformatics/btq033 20110278 PMC2832824

[20] Deatherage DE , Barrick JE . Identification of mutations in laboratory‐evolved microbes from next‐generation sequencing data using breseq. Methods Mol Biol. 2014;1151:165‐188. doi:10.1007/978-1-4939-0554-6_12 24838886 PMC4239701

[21] McKinney W . Data Structures for Statistical Computing in python. In: Proceedings of the Python in Science Conference; 2010.

[22] Hunter JD . Matplotlib: A 2D graphics environment. Comput Sci Eng. 2007;9(3):90‐95. doi:10.1109/MCSE.2007.55

[23] Waskom M . Seaborn: Statistical data visualization. J Open Source Softw. 2021;6(60):3021. doi:10.21105/joss.03021

[24] Kosakovsky Pond SL , Poon AF , Velazquez R , et al. Hyphy 2.5—a customizable platform for evolutionary hypothesis testing using phylogenies. Mol Biol Evol. 2020;37(1):295‐299. doi:10.1093/molbev/msz197 31504749 PMC8204705

[25] Libin PJ , Deforche K , Abecasis AB , Theys K . VIRULIGN: Fast codon‐correct alignment and annotation of viral genomes. Bioinformatics. 2018;35(10):1763‐1765. doi:10.1093/bioinformatics/bty851 PMC6513156 30295730

[26] Smith MD , Wertheim JO , Weaver S , Murrell B , Scheffler K , Kosakovsky Pond SL . Less is more: an adaptive branch‐site random effects model for efficient detection of episodic diversifying selection. Mol Biol Evol. 2015;32(5):1342‐1353. doi:10.1093/molbev/msv022 25697341 PMC4408413

[27] Wickham H , François R , Henry L , Müller K . dplyr: A Grammar of Data Manipulation. R package version 1.0.10. The Comprehensive R Archive Network (CRAN); 2022.

[28] Wickham H . ggplot2: Elegant graphics for data analysis. Springer‐Verlag; 2016. doi:10.1007/978-3-319-24277-4

[29] Stevens LJ , Pruijssers AJ , Lee HW , et al. Mutations in the SARS‐COV‐2 RNA‐dependent RNA polymerase confer resistance to remdesivir by distinct mechanisms. Sci Transl Med. 2022;14(656):eabo0718. doi:10.1126/scitranslmed.abo0718 35482820 PMC9097878

[30] Zhabokritsky A , Mubareka S , Kozak RA , et al. Persistent infection with severe acute respiratory coronavirus virus 2 (SARS‐COV‐2) in a patient with untreated human immunodeficiency virus (HIV). Infect Control Hosp Epidemiol. 2022;44(2):1‐2. doi:10.1017/ice.2022.140 PMC9171057 35591771

