# Supplementary figures and images for: Use of whole genome sequencing to identify low‐frequency mutations in SARS‐CoV‐2 patients treated with remdesivir
Source: Influenza Other Respir Viruses. 2023 Sep 26;17(9):e13179. doi: 10.1111/irv.13179 (PMC10522481; doi:10.1111/irv.13179)

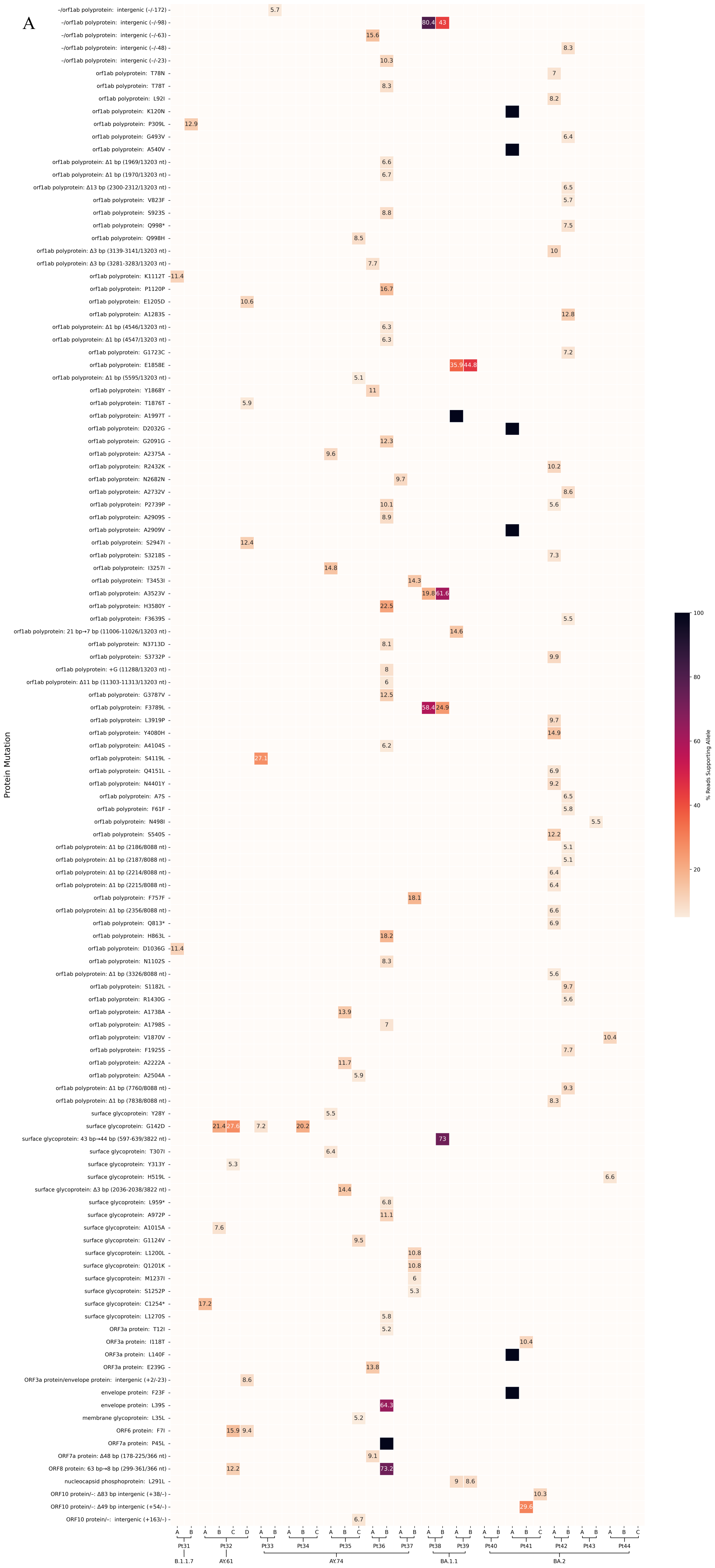

Supplement: Supplementary file 1 — Figure S1a Mutation profile across the whole genome for 35 serial swabs collected from remdesivir treated patients (A) and 74 collected from untreated patients (B). Serial samples are labeled sequentially by date. Mutations were analyzed and called against the reference sequence with the Breseq pipeline. Low frequency minority mutations were retained and percentages are shown. Majority mutations conserved among lineages and within individuals are excluded. Default parameters of Breseq including a minimum 5% polymorphism frequency filter and a polymorphism minimum coverage of 2 were used. [file IRV-17-e13179-s001.pdf]

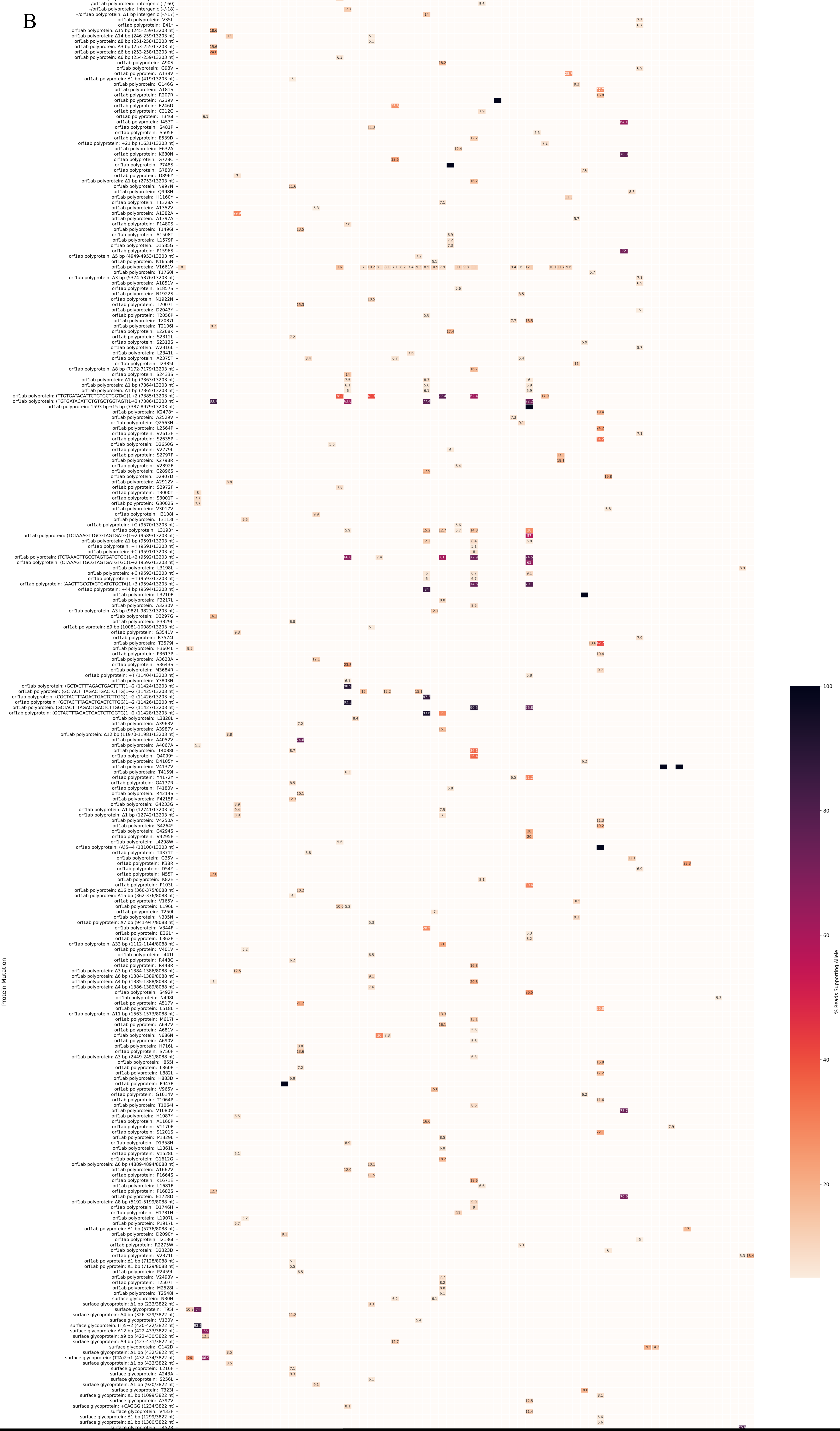

Supplement: Supplementary file 2 — Figure S1b Mutation profile across the whole genome for 35 serial swabs collected from remdesivir treated patients (A) and 74 collected from untreated patients (B). Serial samples are labeled sequentially by date. Mutations were analyzed and called against the reference sequence with the Breseq pipeline. Low frequency minority mutations were retained and percentages are shown. Majority mutations conserved among lineages and within individuals are excluded. Default parameters of Breseq including a minimum 5% polymorphism frequency filter and a polymorphism minimum coverage of 2 were used. [file IRV-17-e13179-s003.pdf]
