# Supplementary material for: Use of whole genome sequencing to identify low‐frequency mutations in SARS‐CoV‐2 patients treated with remdesivir
Source: Influenza Other Respir Viruses. 2023 Sep 26;17(9):e13179. doi: 10.1111/irv.13179 (PMC10522481; doi:10.1111/irv.13179)
Supplement: Supplementary file 3 — Table S1 Cycle threshold for the SAR‐CoV‐2 envelope gene from nasal samples collected from RDV‐treated patients. Table S2 Cycle threshold for the SAR‐CoV‐2 envelope gene from nasal samples collected from patients without RDV treatment. [file IRV-17-e13179-s002.docx]

| **PATIENT** | **SAMPLE** | **Ct (E GENE)** |
| --- | --- | --- |
| Pt31 | A | 19.68 |
| Pt31 | B | 15.90 |
| Pt32 | A | N/A |
| Pt32 | B | 11.51 |
| Pt32 | C | 11.65 |
| Pt32 | D | 18.41 |
| Pt33 | A | 11.00 |
| Pt33 | B | 19.03 |
| Pt34 | A | 19.25 |
| Pt34 | B | 10.79 |
| Pt34 | C | 20.80 |
| Pt35 | A | 16.54 |
| Pt35 | B | 21.35 |
| Pt35 | C | 21.19 |
| Pt36 | A | 22.99 |
| Pt36 | B | 24.50 |
| Pt37 | A | 20.20 |
| Pt37 | B | N/A |
| Pt38 | A | 15.14 |
| Pt38 | B | 21.54 |
| Pt39 | A | 13.37 |
| Pt39 | B | 21.62 |
| Pt40 | A | 16.34 |
| Pt40 | B | 18.97 |
| Pt41 | A | N/A |
| Pt41 | B | 21.13 |
| Pt41 | C | 23.52 |
| Pt42 | A | 25.57 |
| Pt42 | B | 26.89 |
| Pt43 | C | 13.6 |
| Pt43 | D | 20.69 |
| Pt44 | A | N/A |
| Pt44 | B | 14.28 |
| Pt44 | C | 18.00 |

**Supplementary table 1: Cycle threshold for the SAR-CoV-2 envelope gene from nasal samples collected from RDV-treated patients.**

Abbreviations: Ct, cycle threshold; E, envelope

**Supplementary table 2: Cycle threshold for the SAR-CoV-2 envelope gene from nasal samples collected from patients without RDV treatment.**

| **PATIENT** | **SAMPLE** | **Ct (E GENE)** |
| --- | --- | --- |
| Pt01 | A | 20.27 |
| Pt01 | B | 24.79 |
| Pt01 | C | 22.97 |
| Pt02 | A | 22.04 |
| Pt02 | B | 25.31 |
| Pt03 | A | 20.81 |
| Pt03 | B | 23.31 |
| Pt03 | C | 22.29 |
| Pt03 | D | 20.99 |
| Pt04 | A | 14.80 |
| Pt04 | B | 15.31 |
| Pt04 | C | 14.46 |
| Pt04 | D | 13.59 |
| Pt05 | A | 17.93 |
| Pt05 | B | 23.42 |
| Pt05 | C | 25.22 |
| Pt06 | A | 17.4 |
| Pt06 | B | 19.53 |
| Pt07 | A | 12.54 |
| Pt07 | B | 13.97 |
| Pt08 | A | 22.64 |
| Pt08 | B | 23.81 |
| Pt09 | A | 18.44 |
| Pt09 | B | 16.81 |
| Pt09 | C | 22.95 |
| Pt10 | A | 13.76 |
| Pt10 | B | 15.60 |
| Pt10 | C | 19.97 |
| Pt11 | A | 10.45 |
| Pt11 | B | 16.51 |
| Pt11 | C | 17.39 |
| Pt12 | A | 27.07 |
| Pt12 | B | 22.02 |
| Pt12 | C | 26.68 |
| Pt13 | A | 22.50 |
| Pt13 | B | 24.25 |
| Pt14 | A | 13.71 |
| Pt14 | B | 23.64 |
| Pt15 | A | 15.47 |
| Pt15 | B | 22.68 |
| Pt16 | A | 16.40 |
| Pt16 | B | 23.64 |
| Pt17 | A | 13.31 |
| Pt17 | B | 20.29 |
| Pt17 | C | 25.03 |
| Pt18 | A | 17.79 |
| Pt18 | B | 22.89 |
| Pt19 | A | 17.20 |
| Pt19 | B | 22.18 |
| Pt19 | C | 17.70 |
| Pt20 | A | 19.46 |
| Pt20 | B | 21.23 |
| Pt21 | A | N/A |
| Pt21 | B | 30.70 |
| Pt21 | C | 22.92 |
| Pt22 | A | 20.35 |
| Pt22 | B | 20.17 |
| Pt23 | A | 21.81 |
| Pt23 | B | 22.59 |
| Pt24 | A | 12.91 |
| Pt24 | B | 16.26 |
| Pt25 | A | 11.07 |
| Pt25 | B | 21.06 |
| Pt26 | A | 17.47 |
| Pt26 | B | 27.56 |
| Pt27 | A | 15.79 |
| Pt27 | B | 13.01 |
| Pt28 | A | 15.13 |
| Pt28 | B | 22.42 |
| Pt29 | A | 18.65 |
| Pt29 | B | 16.72 |
| Pt30 | A | 21.34 |
| Pt30 | B | 21.25 |

Abbreviations: Ct, cycle threshold; E, envelope
